# Causal relationship of interleukin-6 and its receptor on sarcopenia traits using mendelian randomization

**DOI:** 10.1186/s12937-024-00958-w

**Published:** 2024-05-15

**Authors:** Baixing Chen, Shaoshuo Li, Shi Lin, Hang Dong

**Affiliations:** 1 Department of Development and Regeneration, KU Leuven, Leuven, Belgium; 2 Wuxi Affiliated Hospital of Nanjing University of Chinese Medicine, Wuxi, China; 3 Guangzhou University of Chinese Medicine, Guangzhou, Guangdong Province China; 4 Department of traumatology, The First Affiliated Hospital, Guangzhou University of Chinese Medicine, Guangzhou, Guangdong Province China

**Keywords:** Interleukin 6, IL-6 receptor, Sarcopenia, Genome-wide association studies, Mendelian randomization

## Abstract

**Background:**

Previous research has extensively examined the role of interleukin 6 (IL-6) in sarcopenia. However, the presence of a causal relationship between IL-6, its receptor (IL-6R), and sarcopenia remains unclear.

**Method:**

In this study, we utilized summary-level data from genome-wide association studies (GWAS) focused on appendicular lean mass (ALM), hand grip strength, and walking pace. Single nucleotide polymorphisms (SNPs) were employed as genetic instruments for IL-6 and IL-6R to estimate the causal effect of sarcopenia traits. We adopted the Mendelian randomization (MR) approach to investigate these associations using the inverse variance weighted (IVW) method as the primary analytical approach. Additionally, we performed sensitivity analyses to validate the reliability of the MR results.

**Result:**

This study revealed a significant negative association between main IL-6R and eQTL IL-6R on the left grip strength were − 0.013 (SE = 0.004, *p* < 0.001) and -0.029 (SE = 0.007, *p* < 0.001), respectively. While for the right grip strength, the estimates were − 0.011 (SE = 0.001, *p* < 0.001) and − 0.021 (SE = 0.008, *p* = 0.005). However, no evidence of an association for IL-6R with ALM and walking pace. In addition, IL-6 did not affect sarcopenia traits.

**Conclusion:**

Our study findings suggest a negative association between IL-6R and hand grip strength. Additionally, targeting IL-6R may hold potential value as a therapeutic approach for the treatment of hand grip-related issues.

**Supplementary Information:**

The online version contains supplementary material available at 10.1186/s12937-024-00958-w.

## Introduction

Sarcopenia is a syndrome characterized by a loss of muscle mass and strength, which often leads to functional impairment and adverse outcomes [1]. This condition involves a restructuring of the overall muscle composition, including the transformation of muscle fibers and the infiltration of lipids [2]. As a result, muscle power is diminished, and the risk of falls, mortality, disability, and hospitalization is elevated compared to individuals without sarcopenia [3].

Extensive research has demonstrated that interleukins, particularly interleukin-6 (IL-6), play a critical role in the development of skeletal muscle wasting [4]. They achieve this by activating molecular pathways that disrupt the balance between protein synthesis and catabolism [5]. For example, IL-6 has catabolic effects on muscle proteins [6]. In clinical settings, geriatric patients with acute infection-induced inflammation who received piroxicam, a non steroidal anti-inflammatory drug, showed a decreased level of IL-6 with better muscle performance. Several systematic reviews have consistently shown that elevated levels of inflammatory cytokines are inversely correlated with muscle strength and mass [7–9]. On the other hand, therapeutic strategies that IL-6R antagonists, such as the use of tocilizumab, have shown promise in increasing muscle mass. IL-6 promotes inflammatory responses via the membrane-bound or circulating soluble IL-6R. Two studies demonstrated that administration of anti-mouse IL-6 receptor antibodies improved muscle mass in mice [10, 11]. In a study by Tournadre et al., the use of tocilizumab was shown to have beneficial effects on lean mass without significant changes in fat mass in patients with rheumatoid arthritis [12]. IL-6 and sIL-6R are important factors in the regulation of inflammation, but their effects on muscle mass and function suggest that the relationship for IL-6 and sIL-6R with sarcopenia is complex and may be mediated by other factors. Further research is needed to better understand the mechanisms underlying the association.

Mendelian randomization (MR) is an analytical approach that utilizes germline genetic markers as instrumental variables to assess potential risk factors [13]. The random allocation of genetic variants from parents to offspring during gametogenesis provides protection against confounding factors typically encountered in observational studies and helps to mitigate issues of reverse causation [14]. The growing availability of genetic association data for various traits and diseases has significantly enhanced the utility of MR methods for establishing reliable causal inferences. In particular, the inclusion of genome-wide association study (GWAS) data from large-scale consortia has the potential to enhance the statistical power of MR analyses for detecting causal effects [15]. MR provide a causal inference approach to establish whether the association between IL-6 or sIL6 and sarcopenia is causal or merely a correlation. Therefore, the aim of this study was to comprehensively investigate the causal relation for IL-6 or sIL6 with sarcopenia using a two-sample Mendelian randomization approach, and to determine whether targeting these factors may be a viable approach to preventing or treating muscle loss.

## Method

### Study design

This two-sample Mendelian randomization (MR) study design utilized summary-level data and relied on three key assumptions. In our analysis, we ensured that the instrumental variables (genetic variants) used in the study satisfied three key assumptions. Firstly, these genetic variants showed a strong association with the exposure of interest, as demonstrated by their genome-wide association study *p*-value being less than 5 × 10^− 8^, thus fulfilling the relevance assumption. Secondly, we confirmed that these genetic variants were independent of potential confounders that could influence the relationship between the exposure and outcome, fulfilling the independence assumption. Lastly, we assumed that these genetic variants only affected the outcome through the exposure of interest and not through any other causal pathways, adhering to the exclusion restriction assumption. These assumptions are crucial for valid MR analysis and help ensure the reliability of our results. In short, the MR analysis required that the genetic variants meet these assumptions in order to provide valid causal inferences [16].

### Data sources of exposure

Table S1 presented an overview of the data sources used in this study, including sample sizes and characteristics of the GWAS data sources. The interleukin GWAS data was obtained from Folkersen et al. [17], and adjusted for covariates by accounting for population structure and study-specific parameters. To fulfill the first assumption of MR, we included all single nucleotide polymorphisms (SNPs) that strongly and independently (R^2^ < 0.001) predicted the exposures of interest at genome-wide significance (*P* < 5 × 10^–8^). We also took steps to remove SNPs that showed potential pleiotropic effects, which could introduce confounding in the analysis [18]. To confirm our hypothesis and enhance the integrity of our findings, specific genetic instruments for IL-6 and IL-6R were crafted. Genetic variants were selected within a 150 kb region around the genes encoding IL-6 (ENSG00000136244) and IL-6R (ENSG00000160712), establishing distinct genetic tools aimed at directly influencing these target genes [19]. To assess the strength of the selected genetic predictors for interleukins, we excluded SNPs with an F-statistic below 10. The mean value of the remaining SNPs was then calculated to obtain the estimated F-statistic for each exposure factor. This approach helps evaluate the robustness and reliability of the genetic predictors in our analysis [20].

### Outcomes in GWAS: sarcopenia traits

Summary statistics for sarcopenia traits were extracted from the Pie et al. GWAS [21] and UK Biobank (Neale Lab) [22]. Sarcopenia traits included appendicular lean mass (ALM), hand grip strength and walking pace, as followed: ALM (*n* = 450,243), left grip strength left(*n* = 335,821), right grip strength right (*n* = 335, 842) and walking pace (*n* = 335,349). The ALM data utilized in this study were obtained from the UK Biobank, employing bioelectric impedance analysis (BIA) to assess the combined muscle mass of the arms and legs [23]. Grip strength, which demonstrated a moderate correlation with overall body strength, was selected as a dependable surrogate for measuring whole-body strength [24]. Walking pace, known for its speed, safety, and high reliability, was widely adopted as a practical test for sarcopenia in clinical settings [24].

### MR analysis

In this study, the inverse variance weighted (IVW) method was utilized to assess the causal effects and to evaluate the bi-directional relation between interleukin and sarcopenia traits. To obtain a pooled causal estimate, this method utilizes the Wald ratio of each SNPs on the outcome. In our analysis, we applied a Bonferroni correction by setting a threshold of *P* < 0.0125 (α = 0.05/4 outcomes) to account for multiple comparisons and maintain a stringent level of statistical significance. Nevertheless, if the instrumental variables (IVs) violate the assumption of “no horizontal pleiotropy,” the estimated results obtained through the IVW method may be biased. To account for this potential bias, we performed sensitivity analyses using two additional MR methods. Firstly, we applied the weighted median (WM) method developed by Bowden et al. [25]. This method is robust and produces consistent causal estimates even when more than 50% of the IVs are valid. In addition, we employed MR-Egger regression to assess the presence of unbalanced pleiotropy and significant heterogeneity. It is worth noting that this method typically requires a larger sample size to achieve the same level of precision in estimating the underexposure variatio [26].

### Sensitivity analysis

Horizontal pleiotropy can be a potential issue in MR studies, as it can lead to biased estimates of causal effects. To ensure the robustness of our findings, we conducted additional analyses to detect the presence of pleiotropy and heterogeneity. To evaluate heterogeneity, we employed Cochran’s Q statistic, where a significant result is indicated by a *p*-value less than 0.05. This statistical test allows us to determine if there is substantial heterogeneity among the studies included in our analysis [27]. Additionally, we employed the MR-Egger regression to appraise horizontal pleiotropy, and considered a *P* value less than 0.05 to be significant [28]. If the IVW method result was significant (*P* < 0.05), even if other methods did not show significant results and no pleiotropy or heterogeneity was identified, we considered it as a positive result, as long as the beta values of other methods were in the same direction. All analyses were conducted using R (version 4.1.1) and the R packages “TwosampleMR” [29].

## Result

### The effect of interleukin on sarcopenia traits

In this study, two and three SNPs were available for main IL-6 instrument and eQTL IL-6 instrument, respectively. Seven and eight SNPs were available for main IL-6R instrument and eQTL IL-6R instrument, respectively, all with a genome-wide significance (*p* < 5 × 10^–8^) (Table S2). No causal relationship between IL-6 and sarcopenia traits was observed. Given that there are only two and three SNPs for IL6, a *p*-value of 5 × 10^–5^ was set as the threshold to include more SNPs. However, the same SNPs were identified using this threshold. In IVW analysis, a significant negative associations between IL-6R levels and grip strength was observed. The effect estimates for main IL-6R and eQTL IL-6R on the left grip strength were − 0.013 (SE = 0.004, *p* < 0.001) and -0.029 (SE = 0.007, *p* < 0.001), while forn the right grip strength, the estimates were − 0.011 (SE = 0.001, *p* < 0.001) and − 0.021 (SE = 0.008, *p* = 0.005), as presented in Table 1. However, no causal association for IL-6R with ASM or walking pace was observed. To assess the robustness of our findings, we performed several sensitivity analyses, including the Cochran’s Q test and the MR-Egger intercept test (Table 2). However, these methods were not applicable for main IL-6 due to the limited number of instrumental variables (IVs) available for this analysis.

As IL6 is also a myokine secreted by muscle cells, a reverse MR analysis was performed to identify the effect of sarcopenia traits on interleukin. The results of this analysis, as presented in Table S3, indicated that there were no statistically significant causal effects of these sarcopenia traits on IL-6 or IL-6R. Furthermore, Cochran’s Q tests, detailed in Table S4, revealed *P*-values greater than 0.05, suggesting an absence of heterogeneity among the instrumental variables used in our analysis. These findings suggest that, within the limits of our study design and the genetic instruments employed, sarcopenia traits do not exert a detectable causal influence on IL-6 or IL-6R levels.

**Table 1:** MR results of the causal effect of interleukin on sarcopenia traits

Exposure	Methods	ALM		HGS (left)		HGS (right)		Walking pace	
		β (SE)	*P*	β (SE)	*P*	β (SE)	*P*	β (SE)	*P*
Main IL-6 (n SNPs = 2)	IVW	-0.045 (0.095)	0.63	-0.023 (0.065)	0.72	-0.026 (0.066)	0.70	-0.017 (0.012)	0.15
	WM								
	MR-Egger								
eQTL IL-6 (n SNPs = 3)	IVW	0.006 (0.013)	0.67	-0.020 (0.012)	0.11	-0.027 (0.015)	0.07	-0.001 (0.010)	0.99
	WM	0.007 (0.014)	0.63	-0.022 (0.014)	0.12	-0.032 (0.014)	0.02	0.003 (0.012)	0.81
	MR-Egger	-0.019 (0.082)	0.86	-0.021 (0.087)	0.85	-0.025 (0.110)	0.92	-0.022 (0.055)	0.75
Main IL-6R (n SNPs = 7)	IVW	-0.007 (0.004)	0.10	-0.013 (0.004)	< 0.001	-0.011 (0.004)	< 0.001	-0.005 (0.003)	0.08
	WM	-0.007 (0.005)	0.15	-0.014 (0.004)	< 0.001	-0.013 (0.004)	0.01	-0.003 (0.004)	0.47
	MR-Egger	-0.004 (0.009)	0.70	-0.002 (0.007)	0.82	-0.002 (0.008)	0.85	-0.005 (0.006)	0.48
eQTL IL-6R (n SNPs = 8)	IVW	-0.002 (0.008)	0.05	-0.029 (0.007)	< 0.001	-0.021 (0.008)	0.005	0.002 (0.009)	0.79
	WM	-0.019 (0.010)	0.05	-0.026 (0.010)	0.009	-0.012 (0.010)	0.20	0.007 (0.009)	0.41
	MR-Egger	-0.001 (0.026)	0.96	-0.034 (0.026)	0.82	-0.026 (0.027)	0.36	-0.011 (0.032)	0.75

MR: Mendelian Randomization; ALM: appendicular lean mass; HGS: hand grip strength; IL-6: interleukin-6; IL-6R: interleukin-6 receptor

**Table 2:** Assessment of pleiotropy and heterogeneity in the causal association between interleukin and sarcopenia traits

Exposure	Outcome	Cochran Q		MR-Egger	
		Q value	*P*	Intercept	*P*
Main IL-6R	ALM	6.721	0.24	0.001	0.70
	HSG (left)	8.679	0.12	-0.003	0.21
	HSG (right)	7.515	0.19	-0.004	0.10
	Walking pace	5.428	0.37	-0.001	0.98
eQTL IL-6	ALM	1.500	0.47	0.003	0.81
	HSG (left)	1.781	0.41	0.001	0.99
	HSG (right)	2.886	0.24	-0.001	0.92
	Walking pace	0.626	0.73	0.002	0.75
eQTL IL-6R	ALM	6.238	0.51	-0.002	0.59
	HSG (left)	6.947	0.43	0.001	0.85
	HSG (right)	7.508	0.38	0.001	0.84
	Walking pace	15.580	0.06	0.002	0.69

ALM: appendicular lean mass; HGS: hand grip strength; IL-6: interleukin-6; IL-6R: interleukin-6 receptor

## Disscusion

This MR study demonstrated a significant and negative association between IL-6R and hand grip strength. However, no evidence of an association between IL-6 and sarcopenia traits was found. These findings offer new insights into the impact of interleukins on hand grip strength.

Immune aging is closely associated with the development of sarcopenia, leading to the loss of skeletal muscle mass and function [30]. Inflammatory parameters have been shown to be inversely related to hand grip strength [31], suggesting a potential role of the immune system in skeletal muscle protein metabolism during aging [32]. IL-6, as one of the inflammatory factors, plays a crucial role in modulating muscle anabolism or catabolism in response to tissue damage or infection. Previous meta-analyses have consistently demonstrated a negative association between IL-6 and muscle strength and mass. However, it is important to consider the potential influence of reverse causation bias or confounding factors in these associations [9]. Prolonged exposure to IL-6 has been associated with the promotion of muscle atrophy through the suppression of muscle anabolism and energy homeostasis, as well as the direct induction of muscle catabolism [33]. During the development of sarcopenia, there is an increase in the secretion of inflammatory factors, contributing to a low-grade inflammatory state. Moreover, skeletal muscle mass and function decline with age, accompanied by a decrease in the synthesis and secretion of myogenic inflammatory factors, which disrupts skeletal muscle energy metabolism [34]. However, no relationship between IL-6 and sarcopenia traits was observed in this study. This inconsistency may be due to previous studies that found the associations through observational studies, which are prone to confounding and reverse causation. In contrast, MR analysis can help to overcome these limitations by using genetic variants that are less likely to be influenced by confounding factors [35]. It is also possible that IL-6R has a different biological function and effect on muscle strength compared to IL-6. This may explain why our MR analysis did not find a significant association with sarcopenia traits for IL-6 but for IL-6R. Overall, our findings suggest that the previously reported association between IL-6 and muscle mass may not be causal and that IL-6R may have a direct effect on hand grip strength. However, further studies are needed to confirm these findings and to investigate the underlying biological mechanisms.

Furthermore, the methods used to measure muscle mass can vary between studies, and this can contribute to differences in findings. ALM is a commonly used measure of muscle mass in the context of sarcopenia, but it is not the only measure available [36]. Other measures, such as whole-body muscle mass or muscle cross-sectional area, may be more appropriate in certain contexts. Additionally, the way in which ALM is calculated (i.e., whether it is divided by height, weight, or BMI) can also impact the results.

Our study has several limitations that should be considered when interpreting our findings. First, our analysis was conducted using large-scale GWAS data, which may not represent the entire population. Additionally, the sample size for the IL-6 was relatively small, which may limit the generalizability of your findings. Second, While MR can help to address issues of confounding, there may be additional confounders that were not accounted for in our study. For example, factors such as diet, physical activity, or medication use may influence both interleukin levels and muscle mass. Third, we only focused on IL-6R and IL-6 and did not examine the full range of interleukins that may be relevant to muscle mass. Other interleukins may have different effects on muscle mass, and examining a broader range of interleukins may provide a more complete picture of their relationship with muscle mass.

## Conclusion

This is the first study of which we are aware that investigates potential causal associations for IL-6 and IL-6R with sarcopenia traits using Mendelian Randomization. Our findings indicate that IL-6R is negatively associated with hand grip strength. Further research should aim to identify mechanisms of action and targeting IL-6R may be valuable for treatment.

### Electronic supplementary material

Below is the link to the electronic supplementary material.

Supplementary Material 1

Supplementary Material 2

Supplementary Material 3

Supplementary Material 4

## Data Availability

The datasets analyzed in this study are publicly available summary statistics.
