# Supplementary material for: Causal relationship of interleukin-6 and its receptor on sarcopenia traits using mendelian randomization
Source: Nutr J. 2024 May 15;23:51. doi: 10.1186/s12937-024-00958-w (PMC11094953; doi:10.1186/s12937-024-00958-w)
Supplement: Supplementary file 1 — Supplementary Material 1 [file 12937_2024_958_MOESM1_ESM.docx]

| **Supplementary Table 1**. Data source of the study | | | |
| --- | --- | --- | --- |
|  |  |  |  |
| Traits | Source | Sample size | Ancestry |
| Exposures |  |  |  |
| Main IL-6 | Folkersen et al GWAS [16] | Over 30,000 individuals of European descent | European |
| Main IL-6R | Folkersen et al GWAS [16] | Over 30,000 individuals of European descent | European |
| eQTL IL-6 | eQTLGen and GTEx portal release V8 | 31,684 | European |
| eQTL IL-6R | eQTLGen and GTEx portal release V8 | 31,684 | European |
| Outcomes |  |  |  |
| ALM | Pie et al GWAS [19] | 450,243 | European |
| HSG (right) | Neale Lab | 335,842 | European |
| HSG (left) | Neale Lab | 335,821 | European |
| Walking pace | Neale Lab | 335,349 | European |
| ALM: appendicular lean mass; HGS: hand grip strength; IL-6: interleukin-6; IL-6R: interleukin-6 receptor | | | |
