# Supplementary material for: Causal relationship of interleukin-6 and its receptor on sarcopenia traits using mendelian randomization
Source: Nutr J. 2024 May 15;23:51. doi: 10.1186/s12937-024-00958-w (PMC11094953; doi:10.1186/s12937-024-00958-w)
Supplement: Supplementary file 2 — Supplementary Material 2 [file 12937_2024_958_MOESM2_ESM.docx]

| Table S2. Instrument variables of IL-6 and IL-6R | | | | | | | |
| --- | --- | --- | --- | --- | --- | --- | --- |
| SNP | Effect_allele | Other_allele | Effect allele frequency | Beta | SE | P-value | F statistics |
| **Main IL-6** |  |  |  |  |  |  |  |
| rs2228145 | C | A | 0.379 | 0.17 | 0.01 | 3.34E-45 | 233.72 |
| rs4959106 | C | T | 0.463 | 0.08 | 0.01 | 2.37E-09 | 64.68 |
| **IL6 150 kb, eQTL** |  |  |  |  |  |  |  |
| rs11766273 | A | G | 0.081 | 0.14 | 0.02 | 1.16E-10 | 25.8 |
| rs11766947 | C | T | 0.275 | 0.11 | 0.01 | 6.42E-17 | 69.9 |
| rs4621699 | G | A | 0.228 | -0.08 | 0.01 | 4.13E-09 | 78.6 |
| **Main IL-6R** |  |  |  |  |  |  |  |
| rs11264224 | C | A | 0.181 | 0.45 | 0.01 | 1.00E-200 | 1350.41 |
| rs116141616 | A | G | 0.027 | -0.57 | 0.04 | 9.27E-48 | 210.80 |
| rs1218552 | A | G | 0.646 | -0.05 | 0.01 | 2.21E-08 | 31.38 |
| rs61805738 | A | G | 0.036 | 0.30 | 0.03 | 6.62E-21 | 87.86 |
| rs61811421 | T | C | 0.244 | -0.25 | 0.01 | 1.19E-98 | 445.91 |
| rs72633650 | C | T | 0.138 | 0.35 | 0.01 | 1.17E-168 | 762.27 |
| rs72702900 | A | T | 0.040 | 0.20 | 0.03 | 2.42E-12 | 49.15 |
| **IL6R 150 kb, eQTL** |  |  |  |  |  |  |  |
| rs10908839 | G | C | 0.783 | -0.167 | 0.01 | 4.09E-34 | 250.3 |
| rs146563764 | A | G | 0.017 | 0.173 | 0.03 | 3.45E-08 | 150.8 |
| rs4576655 | T | C | 0.418 | -0.085 | 0.01 | 4.23E-12 | 198.0 |
| rs4845626 | T | G | 0.168 | -0.133 | 0.02 | 1.12E-17 | 122.1 |
| rs57569414 | A | C | 0.121 | -0.100 | 0.02 | 2.79E-08 | 57.8 |
| rs58229059 | A | G | 0.009 | -0.323 | 0.04 | 1.15E-13 | 98.9 |
| rs7543174 | T | C | 0.822 | 0.112 | 0.02 | 6.87E-13 | 45.5 |
| rs79480105 | A | G | 0.044 | 0.193 | 0.03 | 3.67E-10 | 101.2 |

IL-6: interleukin-6; IL-6R: interleukin-6 receptor; eQTL: expression quantitative trait loci
