# Supplementary material for: Causal relationship of interleukin-6 and its receptor on sarcopenia traits using mendelian randomization
Source: Nutr J. 2024 May 15;23:51. doi: 10.1186/s12937-024-00958-w (PMC11094953; doi:10.1186/s12937-024-00958-w)
Supplement: Supplementary file 3 — Supplementary Material 3 [file 12937_2024_958_MOESM3_ESM.docx]

| Table S3. The effect of sarcopenia traits on interleukin | | | | | | | | | |
| --- | --- | --- | --- | --- | --- | --- | --- | --- | --- |
|  |  | Exposure | | | | | | | |
| Outcome | Methods | ALM | | HGS (left) | | HGS (right) | | Walking pace | |
|  |  | β (SE) | P | β (SE) | P | β (SE) | P | β (SE) | P |
| Main IL-6 | SNP | 650 |  | 85 |  | 99 |  | 29 |  |
|  | IVW | 0.008 (0.028) | 0.76 | -0.192 (0.114) | 0.09 | -0.150 (0.107) | 0.16 | -0.505 (0.258) | 0.05 |
|  | WM | -0.008 (0.045) | 0.84 | -0.210 (0.169) | 0.21 | -0.226 (0.156) | 0.15 | -0.508 (0.347) | 0.14 |
|  | MR-Egger | -0.011 (0.065) | 0.86 | -0.025 (0.479) | 0.59 | 0.030 (0.423) | 0.94 | -0.396 (1.229) | 0.75 |
| eQTL IL-6 | SNP | 94 |  | 16 |  | 19 |  | 6 |  |
|  | IVW | 0.038 (0.048) | 0.44 | 0.055 (0.255) | 0.83 | -0.117 (0.220) | 0.59 | -0.238 (0.525) | 0.65 |
|  | WM | 0.089 (0.076) | 0.24 | 0.375 (0.324) | 0.25 | 0.330 (0.302) | 0.28 | 0.355 (0.680) | 0.60 |
|  | MR-Egger | 0.250 (0.107) | 0.02 | -0.449 (1.041) | 0.67 | -0.695 (0.749) | 0.37 | -3.471 (1.589) | 0.09 |
| IL-6R | SNP | 651 |  | 86 |  | 100 |  | 30 |  |
|  | IVW | -0.032 (0.021) | 0.14 | -0.005 (0.090) | 0.95 | 0.103 (0.085) | 0.23 | -0.094 (0.198) | 0.67 |
|  | WM | -0.007 (0.034) | 0.84 | 0.089 (0.129) | 0.49 | 0.091 (0.126) | 0.47 | -0.301 (0.272) | 0.27 |
|  | MR-Egger | -0.006 (0.005) | 0.23 | 0.206 (0.371) | 0.58 | 0.033 (0.337) | 0.92 | -0.678 (0.949) | 0.48 |
| eQTL IL-6R | SNP | 95 |  | 16 |  | 19 |  | 6 |  |
|  | IVW | -0.025 (0.048) | 0.61 | -0.214 (0.955) | 0.82 | -0.103 (0.085) | 0.54 | -0.113 (0.506) | 0.82 |
|  | WM | -0.02 (0.082) | 0.74 | -0.144 (0.325) | 0.66 | -0.079 (0.306) | 0.80 | -0.287 (0.65) | 0.66 |
|  | MR-Egger | 0.046 (0.106) | 0.67 | 0.222 (0.955) | 0.82 | 0.561 (0.741) | 0.46 | -0.865 (1.589) | 0.62 |
| MR: Mendelian Randomization; ALM: appendicular lean mass; HGS: hand grip strength; IL-6: interleukin-6; IL-6R: interleukin-6 receptor; IVW: inverse variance weighted; WM: Weighted median | | | | | | | | | |
