# Supplementary material for: Causal relationship of interleukin-6 and its receptor on sarcopenia traits using mendelian randomization
Source: Nutr J. 2024 May 15;23:51. doi: 10.1186/s12937-024-00958-w (PMC11094953; doi:10.1186/s12937-024-00958-w)
Supplement: Supplementary file 4 — Supplementary Material 4 [file 12937_2024_958_MOESM4_ESM.docx]

| Table S4 Assessment of pleiotropy and heterogeneity in the causal association for sarcopenia traits on interleukin | | | | | |
| --- | --- | --- | --- | --- | --- |
| Outcome | Outcome | Cochran Q |  | MR-Egger |  |
|  |  | Q value | *P* | Intercept | *P* |
| IL-6 | ALM | 729.8 | 0.14 | 0.0004 | 0.74 |
|  | HSG (left) | 78.7 | 0.64 | 0.0009 | 0.89 |
|  | HSG (right) | 91.7 | 0.66 | -0.0025 | 0.66 |
|  | Walking pace | 21.5 | 0.80 | -0.0011 | 0.93 |
|  |  |  |  |  |  |
| IL-6R | ALM | 698.1 | 0.10 | 0.0007 | 0.54 |
|  | HSG (left) | 84.8 | 0.42 | -0.0029 | 0.56 |
|  | HSG (right) | 105.2 | 0.32 | 0.0009 | 0.83 |
|  | Walking pace | 19.4 | 0.88 | 0.006 | 0.53 |
| ALM: appendicular lean mass; HGS: hand grip strength; IL-6R: interleukin-6 receptor | | | | | |
